# Identification of Novel Adipokines in the Joint. Differential Expression in Healthy and Osteoarthritis Tissues

**DOI:** 10.1371/journal.pone.0123601

**Published:** 2015-04-08

**Authors:** Javier Conde, Morena Scotece, Vanessa Abella, Rodolfo Gómez, Verónica López, Rosa Villar, Miguel Hermida, Jesús Pino, Juan Jesús Gómez-Reino, Oreste Gualillo

**Affiliations:** 1 SERGAS (Servizo Galego de Saude) and IDIS (Instituto de Investigación Sanitaria de Santiago), the NEIRID Lab (Neuroendocrine Interactions in Rheumatology and Inflammatory Diseases), Research Laboratory 9, Santiago University Clinical Hospital, Santiago de Compostela, Spain; 2 Department of Molecular and Cellular Biology, University of Coruña (UDC), A Coruña, Spain; 3 SERGAS (Servizo Galego de Saude), Division of Orthopaedics Surgery and Traumatology, Santiago University Clinical Hospital, Santiago de Compostela, Spain; 4 University of Santiago de Compostela, Department of Medicine and SERGAS (Servizo Galego de Saude) and IDIS (Instituto de Investigación Sanitaria de Santiago), Division of Rheumatology, Santiago University Clinical Hospital, Santiago de Compostela, Spain; Queen Mary University of London, UNITED KINGDOM

## Abstract

**Objectives:**

Emerging data suggest that several metabolic factors, released mainly by white adipose tissue (WAT) and joint tissues, and collectively named adipokines, might have a role in the pathophysiology of OA. Recently, novel adipokines such as SERPINE2, WISP2, GPNMB and ITIH5 have been identified in WAT. The main goal of this study was to analyse the expression of these novel adipokines in synovium, infrapatellar fat pad and chondrocytes and to compare the expression of these molecules in healthy and OA tissues.

**Methods:**

Synovial tissues, infrapatellar fat pad and chondrocytes were obtained from 36 OA patients (age 52–85; mean BMI 28.9) who underwent total knee replacement surgery. Healthy synovial tissues and infrapatellar fat pad were obtained from 15 traumatic knee patients (age 23–53; mean BMI 23.5). mRNA and protein expression were determined by qRT-PCR and western blot analysis respectively.

**Results:**

All the novel adipokines, matter of our study, are expressed in OA synovium, infrapatellar fat pad and chondrocytes. Moreover, we detected a differential expression of SERPINE2 and ITIH5 in OA synovial tissues as compared to healthy samples. Finally, we also observed an increased expression of WISP2 in OA infrapatellar fat pad in comparison to healthy controls.

**Conclusions:**

In this study we demonstrated for the first time the expression of four novel adipokines in different joint tissues and how these molecules are differentially expressed in healthy and OA joint tissues.

## Introduction

Osteoarthritis (OA) is one of the most common form of arthritis and a major cause of pain and disability in adult population. Although, OA was first considered a disorder of the articular cartilage, nowadays it is generally recognized that OA affects all joint tissues, including synovium, ligaments, tendons, muscle and subchondral bone [1]. Several risk factors contribute to osteoarthritis development, including sex, age, mechanical factors or obesity, among others. Due to the increased fat mass, obesity enhances mechanical stress in weight bearing joints, but also contributes to joint tissues degeneration by producing and releasing a plethora of factors called adipokines [2]. Adipose tissue is currently considered a very active endocrine organ able to secrete many factors which could participate in the pathophysiology of OA [2]. Noteworthy, most of these molecules are produced and secreted also by joint cell populations such as chondrocytes and/or synovial fibroblasts [3,4].

Very recently the expression of different genes involved in cell differentiation and turnover of extracellular matrix have been identified in adipocytes [5,6]. Serpin peptidase inhibitor, clade E member 2 (SERPINE2); WNT1 inducible signalling pathway protein 2 (WISP2); glycoprotein (transmebrane) nmb (GPNMB) and inter-alpha-trypsin inhibitor heavy chain family, member 5 (ITIH5) have been characterized as potential new adipokines [5,6]. All these genes are up-regulated in obesity [6]. In addition, few functional studies postulated the involvement of these new adipokines in different obesity-related processes [7,8].

Previously, we have demonstrated that chondrocytes, synovial tissues and infrapatellar fat pad (IPFP) expressed efficiently several adipokines, most of them with pro-inflammatory features [3,9].

Thus, in the present study we aimed to analyze the constitutive expression of these new adipokines (SERPINE2, WISP2, GPNMB and ITIH5) in different joint tissues such as chondrocytes, synovium and infrapatellar fat pad. Moreover, we assessed and compared the expression of these factors in healthy and OA synovial tissues and in the infrapatellar fat pad.

## Methods

### Patients and samples

This study was conducted with the approval of the Santiago University Clinical Hospital Ethics Committee, approval Number 2014/310. Participants provide their written informed consent to participate in this study. Samples were extracted from thirty six OA patients (age 52–85; mean BMI 28.9) who underwent total knee joint replacement. Fifteen healthy controls (age 23–53; mean BMI 23.5) with traumatic knee lesions (no clinical history of osteoarthritis diseases) were also included in the study.

Collection of samples was conducted with the approval of the Santiago University Clinical Hospital Ethics Committee. Synovial tissues and infrapatellar fat pads were collected, washed and stored at -80°C. Cartilage samples were used to obtain human primary chondrocytes cultures.

### Cell culture

Human primary chondrocytes culture was developed as previously described [3]. Briefly, Human chondrocytes were cultured in DMEM/Ham’s F12 medium supplemented with 10% of fetal bovine serum, L-glutamine, and antibiotics (50 units/ml penicillin and 50 μg/ml streptomycin).

RNA isolation and real-time reverse transcription–polymerase chain reaction (RT-qPCR) mRNA levels were determined using SYBR-green based quantitative PCR (qPCR). Briefly, mRNA from synovial tissues, infrapatellar fat pad and chondrocytes was extracted using TRIzol (Life Technologies, NY, USA) and NucleoSpin kit according to the manufacturer’s instructions. The mRNA was reverse-transcribed (RT) using a SABiosciences First Strand Kit. After the RT reaction, qPCR analysis was performed with a SABiosciences Master Mix and specific PCR primers for: human SERPINE2 (156 bp, PPH08354A, reference position 1101, GenBank accession no. NM_006216.3); human WISP2 (123 bp, PPH00981B, reference position 1257, GenBank accession no. NM_003881.2); human GPNMB (110 bp, PPH18941A, reference position 1406, GenBank accession no. NM_002510.2); human ITIH5 (149 bp, PPH14913A, reference position 1843, GenBank accession no. NM_030569.6); human GAPDH (175 bp, PPH00150E, reference position 1287, GenBank accession no. NM_002046.3). The data were calculated, using the comparative (ΔΔCt) method and the MxPro software (Stratagene, CA, USA), as the ratio of each gene to the expression of the housekeeping gene. Data are shown as mean ± s.e.m (error bars) of the independent samples and represented as fold-change vs. controls.

### Western blot analysis

Small pieces of frozen tissues were placed into 1.5 mL centrifuge tubes and then homogenized by using a Turrax homogenizer (IKA, Germany) in lysis buffer for protein extraction (10 mM Tris/HCl, pH 7.5, 5 mM EDTA, 150 mM NaCl, 30 mM sodium pyrophosphate, 50 mM sodium fluoride, 1 mM sodium orhtovanadate, 0.5% Triton X-100, 1mM PMSF, protease inhibitor cocktail). Tissues lysates were obtained by centrifugation at 14.000g for 20 min at 4°C.

Equal amount of protein were subjected to 10% sodium dodecyl sulphate polyacrylamide gel electrophoresis and transferred to a polyvinylidene difluoride transfer membrane (Immobilon-P transfer membrane, Millipore, MA) using a Trans-Blot semi-dry transfer cell (BioRad, CA, USA). Blots were incubated with the appropriate antibody (anti-SERPINE2 1:1000, R&D Systems, MN, USA; anti-ITIH5 1:1000, ABCAM, UK; anti-WISP2 1:1000, Abnova, Taiwan). Immunoblots were visualized with Immobilon Western Detection kit (Millipore, MA) using horseradish peroxidase-labeled secondary antibody. To confirm equal loading for each sample, membranes were incubated with stripping buffer (100 mM β-mercaptoethanol, 2% SDS, 62.5 mM Tris-HCl pH 6.7) and re-blotted with anti-β-actin antibody (Sigma, MO, USA). Images were captured and analyzed with an EC3 imaging system (UVP, CA, USA).

### Agarose gel electrophoresis

Amplicons of the different genes were electrophoresed on 2% agarose gel and stained with ethidium bromide.

### Statistical analysis

Data are reported as mean ± S.E.M. (error bars) of the independent samples. Statistical analyses were performed by ANOVA followed by unpaired *t*-test and Student-Newman-Keuls test, using the GraphPad Prism 4 software, with *p* values <0.05 considered significant.

## Results

**Fig 1:**
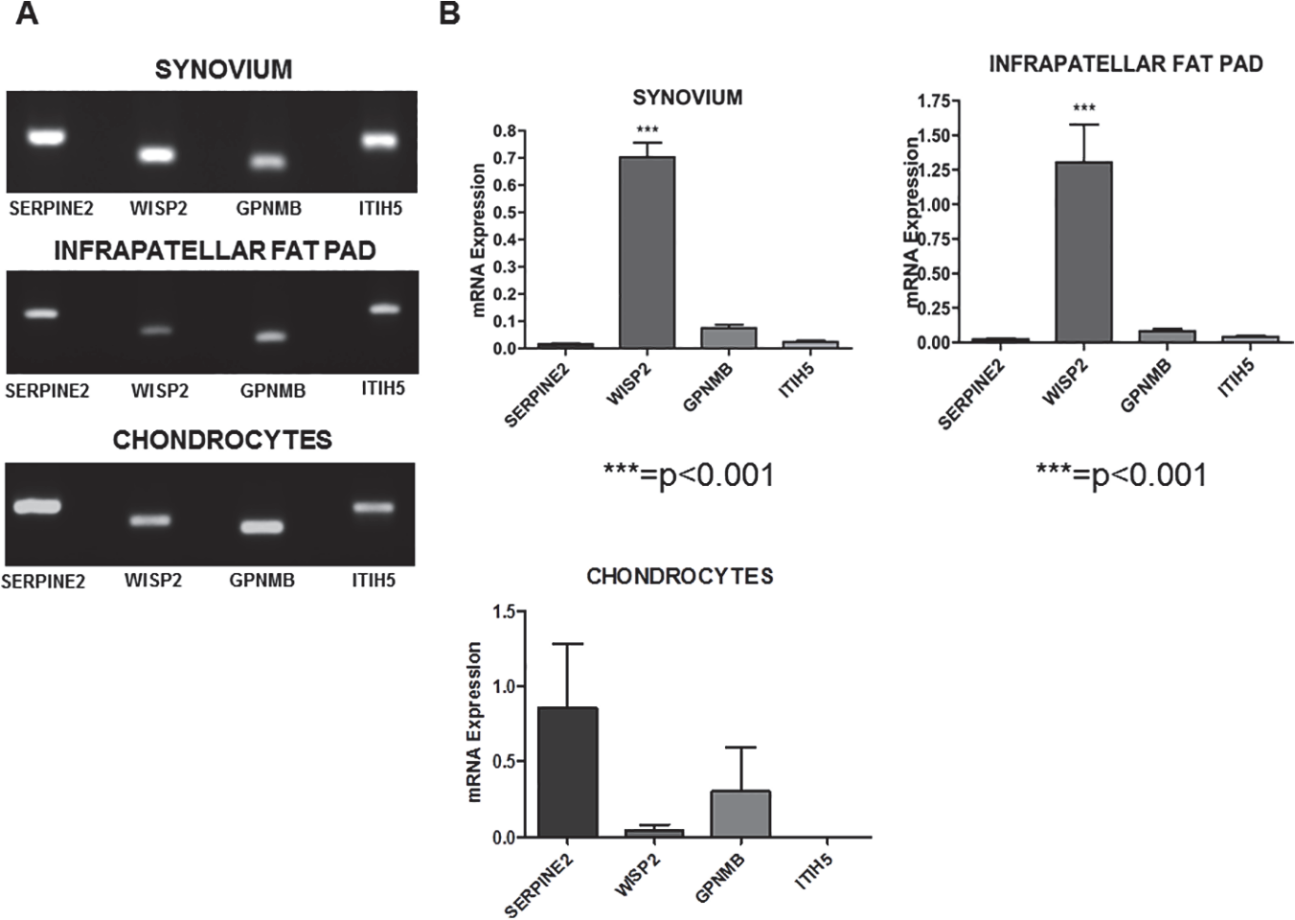
SERPINE2, WISP2, GPNMB and ITIH5 expression evaluated by real-time reverse transcription PCR (qRT-PCR) in synovia, IPFP and chondrocytes obtained from OA patients. A. Amplicons were electrophoresed on 2% agarose gel and stained with ethidium bromide. B. Determination of the basal mRNA expression of SERPINE2, WISP2, GPNMB and ITIH5 in synovial tissues, IPFP and chondrocytes of 30 OA patients. The results were expressed using the ΔΔC_t_ data analysis method. Statistical analysis was performed using One-way ANOVA followed by Newman-Keuls Multiple Comparison Test. P value *** = p<0.001 WISP2 vs SERPINE2; GPNMN; ITIH5.

Constitutive expression of SERPINE2, WISP2, GPNMB and ITIH5 in synovium, infrapatellar fat pad and chondrocytes from OA patients. As shown in Fig 1A, we detected the mRNA expression of all the adipokines in the OA synovial tissues and in the IPFPs. These genes displayed a similar expression pattern in both tissues, being WISP2 the most expressed adipokine (Fig 1A).

To note, OA chondrocytes also expressed SERPINE2, WISP2, GPNMB and ITIH5 (Fig 1A). Quantitative expression of SERPINE2, WISP2, GPNMB and ITIH5 mRNAS in the different tissues are reported in Fig 1B.

**Fig 2:**
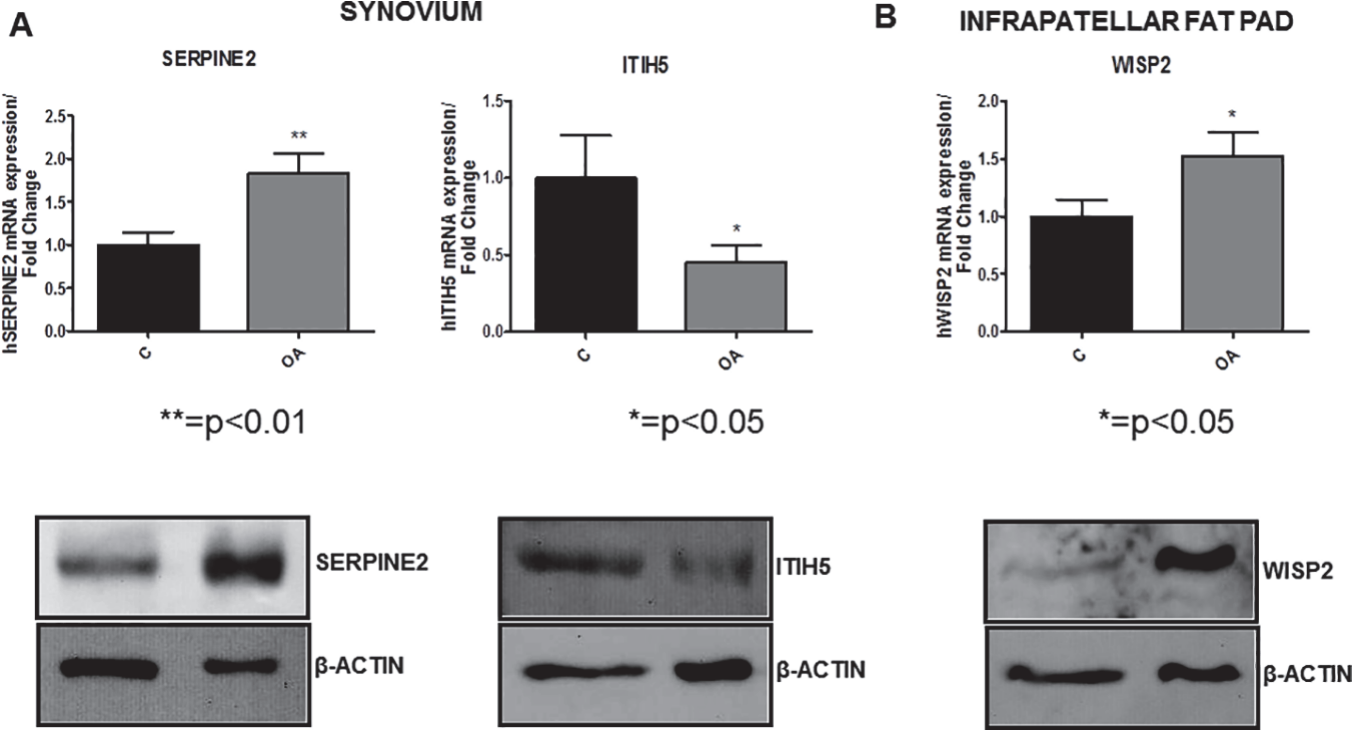
Determination of SERPINE2, WISP2 and ITIH5 mRNA and protein expression by quantitative real-time PCR and western blot. A. PCR results were shown in fold change, where black bars represent the mRNA expression of SERPINE2 and ITIH5 in 10 different synovial tissues obtained from healthy donors. Grey bars represent the mRNA expression of SERPINE2 and ITIH5 in 30 different synovial tissues obtained from OA patients. B. PCR results were shown in fold change, where black bars represent the mRNA expression of WISP2 in IPFP obtained from 10 healthy donors. Grey bars represent the mRNA expression of WISP2 in IPFP obtained from 30 OA patients. Statistical analysis was performed using unpaired *t*-test. P value * = p<0.05; ** = p<0.01 C vs OA.

Comparison between healthy and OA synovium. As shown in Fig 2A, SERPINE2 mRNA and protein expression was significantly up-regulated in OA synovial tissues in comparison to healthy samples. On the other hand, ITIH5 expression was decreased in OA tissues (Fig 2A). However, we did not observe any variation on WISP2 or GPNMB expression between OA and healthy synovial tissues (data not shown).

A, B low panels. Determination of SERPINE2, ITIH5 and WISP2 protein expression by western blot. A representative western blot of five different experiments, using five healthy and five OA samples was shown. β-actin was used to confirm an equal loading for each sample.

Comparison between healthy and OA infrapatellar fat pad. Among all the analyzed adipokines, we only found differences in the expression of WISP2, which was up-regulated in OA IPFP compared to healthy IPFP (Fig 2B).

We did not detect any variation on SERPINE2, GPNMB and ITIH5 expression between healthy and OA samples (data not shown).

## Discussion

Osteoarthritis is considered a disease of the whole joint, which is characterized by articular cartilage degradation, synovial inflammation, ligaments degeneration, but also by bone and muscle alterations [1]. Obesity is considered a relevant risk factor for OA development and progression, not only for the increased mechanical stress to which obese individuals joints are subjected. Also altered adipokine´s secretion, most of them with pro-inflammatory features, due to a dysfunctional adipose tissue contributes to the known chronic low grade inflammatory status presented in obese people, which in turn could affect joint tissues homeostasis [2]. Previously, our group and others have demonstrated the presence of several adipose tissue-derived factors in the OA joint. For instance, leptin and chemerin, positively correlated with the severity of osteoarthritis [9–11]. Actually, the local production by joint tissues has been postulated as an important source of these adipokines as well as other inflammatory mediators [9,12–14]. Therefore, the alteration of their secretion pattern during OA could impact cartilage and synovium homeostasis. In fact, IPFPs and synovial fibroblasts, exposed to pro-inflammatory cytokines such as IL-1β, released large amounts of pro-inflammatory mediators, including prostaglandinE_2_, TNF-α or IL-6, and adipokines such as leptin [15]. Taken together, all these observations suggest that IPFP and synovium are highly active tissues within the joint, able to produce multiple pro-inflammatory factors, which in turn can participate in the initiation and perpetuation of synovitis and OA symptoms.

Very recently novel molecules have been identified in WAT and they are supposed to be involved in cell differentiation and extracellular matrix homeostasis (SERPINE2, WISP2, GPNMB and ITIH5) [5,6]. In this study we sought to analyse the expression of these novel factors in healthy and OA joint tissues, in order to test their potential modulation in OA.

First, we identified the expression of SERPINE2, WISP2, GPNMB and ITIH5 in synoviums and IPFPs of OA patients. All these genes were also detected in OA chondrocytes. Although the expression of WISP2 and GPNMB in OA chondrocytes has been already described in previously published observations [16,17], our study demonstrates, for the first time, the expression of SERPINE2 and ITIH5 in human chondrocytes. Similarly, we also described for the first time the expression of SERPINE2, WISP2, GPNMB and ITIH5 in the synovial tissues and in the IPFPs of OA patients. Our data are partially in agreement with those observed by Tanaka et al., which reported the expression of WISP2 by OA synovial tissues. However, these authors only detected WISP2 expression in some of the analyzed synovial tissues [18], whereas, we found WISP2 expression in all the synovial tissues tested so far.

In order to know whether these new adipokines could be modulated in OA, we compared their expression in both healthy and OA tissues. As shown previously, we observed a differential expression of SERPINE2 and ITIH5 in the synovial tissues from OA patients and healthy controls. During OA progression, synovial tissues may have histological and morphological changes (synovitis) [19]. Although, the exact mechanism that leads to the synovitis in OA is still unclear, pro-inflammatory adipokines, cytokines, chemokines and other molecular products of extracellular matrix degradation were proposed to be at play [19]. Noteworthy, it has been reported that SERPINE2 was able to reduce plasminogen-induced degradation of muscle extracellular matrix. It was also involved in the regulation of the expression of certain metalloproteinases in glioma cell lines [20,21]. In line with this, ITIH family proteins are known to play a role in extracellular matrix stabilization of different cell types [22,23]. Collectively, these evidences, together with our results, suggest that SERPINE2 and ITIH5 might be involved in the histological alterations occurred in the synovial tissues during OA.

In the last years, the pathophysiological role of the IPFP has gained relevant consideration as an active periarticular tissue able to secrete several soluble factors, which could contribute to the pathogenesis of OA [4,9,13]. Actually, our group, very recently, demonstrated that IPFPs from OA patients expressed higher levels of leptin and chemerin in comparison to healthy tissues [9], suggesting that the IPFP may be a source of factors that impact joint tissues [9–11]. In addition, microarray analysis of IPFPs from end-stage OA patients showed an increased expression of different adipokines such as leptin or adiponectin as compared to early-stage OA patients tissues [24], reinforcing the concept of the existence of an altered adipokine secretion pattern in OA joint tissues.

In the present study, we observed high expression of WISP2 in OA IPFPs. WISP2 is a direct target gene of the WNT signaling pathway and it has been reported that different members of the WNT family could participate in the progression of rheumatoid arthritis [25]. Actually, WISP2 likely plays a role in inflammatory arthritis and cell differentiation [7,18]. So, the observation that differential expression of WISP2 observed in our study might have some repercussion on OA progression would seen to make sense

In conclusion, we detected the expression of four new adipokines in cartilage, synovium and IPFP, being some of them differentially expressed in OA tissues compared to healthy samples. Our results suggest a potential involvement of these novel identified adipokines in osteoarthritis. However, further studies are needed to determine the precise mechanism/s by which these molecules may contribute to the onset and progression of osteoarthritis.

### Ethics approval

This study was conducted with the approval of the Santiago University Clinical Hospital Ethics Committee.
